# Post-tuberculosis airway disease: A population-based cohort study of people immigrating to British Columbia, Canada, 1985–2015

**DOI:** 10.1016/j.eclinm.2021.100752

**Published:** 2021-02-26

**Authors:** C. Andrew Basham, Mohammad E. Karim, Victoria J. Cook, David M. Patrick, James C. Johnston

**Affiliations:** a School of Population and Public Health, University of British Columbia, Vancouver, Canada; b British Columbia Centre for Disease Control, Vancouver, Canada; c Centre for Health Evaluative and Outcome Sciences, University of British Columbia, Vancouver, Canada; d Department of Medicine, University of British Columbia, Vancouver, Canada

**Keywords:** Tuberculosis, Respiratory tract diseases, COPD, Asthma, British Columbia, Canada, Cohort study, Survival analysis, Propensity scores

## Abstract

**Background:**

Current epidemiological evidence of post-TB airway disease is largely cross-sectional and derived from high-TB-incidence settings. We present the first cohort study of post-TB airway disease in a low-TB-incidence setting.

**Aims:**

(1) analyze the risk of airway disease by respiratory TB, (2) assess potential unmeasured confounding between TB and airway disease, and (3) investigate TB effect measure modification.

**Methods:**

A population-based cohort study using healthcare claims data for immigrants to British Columbia (BC), Canada, 1985–2015. Airway disease included chronic airway obstruction, asthma, bronchitis, bronchiolitis, and emphysema. Respiratory TB was defined from TB registry data. Cox proportional hazards (PH) regressions were used to analyze time-to-airway disease by respiratory TB. Sensitivity analyses included varying definitions of TB and airway disease. Potential unmeasured confounding by smoking was evaluated by E-value and hybrid least absolute shrinkage and selection operator (LASSO)-high-dimensional propensity score (hdPS).

**Findings:**

In our cohort (*N* = 1 005 328; n_TB_=1141) there were 116 840 incident cases of airway disease during our 30-year study period (10.43 per 1,000 person-years of follow-up), with cumulative incidence of 42·5% among respiratory TB patients compared with 11·6% among non-TB controls. The covariate-adjusted hazard ratio (aHR) for airway disease by respiratory TB was 2·08 (95% CI: 1·91–2·28) with E-value=3·58. The LASSO-hdPS analysis produced aHR=2·26 (95% CI: 2·07–2·47).

**Interpretation:**

A twofold higher risk of airway disease was observed among immigrants diagnosed with respiratory TB, compared with non-TB controls, in a low-TB-incidence setting. Unmeasured confounding is unlikely to explain this relationship. Models of post-TB care are needed.

**Funding:**

Canadian Institutes of Health Research.

**Research in context**

**Evidence before this study**

Four systematic reviews of post-tuberculosis (post-TB) airway disease have been published with a consensus that there is an increased risk of respiratory impairment after TB treatment completion. However, these reviews also identified limitations in the published literature, particularly the use of cross-sectional designs, small samples, and unmeasured confounding. A dearth of literature describing post-TB airway disease in low-TB-incidence settings further limits the generalizability of review findings. A cross-sectional study from Finland examined COPD among people with past TB, and excluded people with pre-existing asthma, finding an adjusted odds ratio of 2.68 (95% CI: 2.00–3.61) and is the most comparable study to the present study.

**Added value of this study**

This is the first cohort study of post-TB airway disease in a low-TB-incidence setting, and one of a few able to remove people with pre-existing airway disease. We demonstrate a twofold increased risk of airway disease by respiratory TB compared to controls. Cumulatively, 42% of respiratory TB patients in our cohort, who did not have airway disease prior to cohort entry, developed airway disease during follow-up. Multiple sensitivity analyses suggest that unmeasured confounding by smoking is unlikely to account for increased risk of airway disease by respiratory TB.

**Implications of all the available evidence**

Combined with existing evidence, a globally increased risk of post-TB airway disease is implied, warranting models of care development for post-TB airway diseases in low-TB-incidence settings.

Alt-text: Unlabelled box

## 1 Introduction

With an estimated 155 million TB survivors alive globally in 2020 [48], post-tuberculosis (post-TB) health has come to the forefront of international TB discourse and has become a research priority [1], [2], [3], [4]. The elevated risk of post-TB respiratory disease has been demonstrated in TB survivors, but the evidence is currently limited in terms of study designs, small sample sizes, lack of studies from low-TB-incidence settings, and potential confounding bias in published studies [5], [6], [7], [8].

Despite limitations in the epidemiologic literature, it is apparent that post-TB airway disease is an important problem [[5], [6], [7],[9], [10], [11], [12], [13], [14]]. While resource constraints in high-TB-incidence settings limit the practicality of systematically assessing airway disease in TB survivors, high resource nations in North America and Western Europe have also not addressed this issue, even with simple investigations such as spirometry or exercise testing [15,16]. In fact, few epidemiological studies to date have examined post-TB airway disease risk in low-TB-incidence settings, and none have used cohort designs [6,7]. These studies have been unable to control for pre-existing airway disease [7]. Other studies, from higher TB incidence settings, have also primarily used cross-sectional designs [6,7]. Limited study of post-TB airway disease in low-TB-incidence settings raises questions about the generalizability of systematic review findings, including the relative and absolute risk of airway disease attributable to respiratory TB, in low-TB-incidence settings.

To the best of our knowledge, this paper presents the first cohort study of post-TB airway disease risk in a low-TB-incidence setting [5], [6], [7]. Our primary aim was to analyze the risk of airway disease in respiratory TB survivors compared with non-TB controls in British Columbia (BC), Canada. Our secondary aim was to assess the potential impact of unmeasured confounding by smoking, for which information is frequently absent from health administrative datasets, and is considered a critical potential confounder [6]. Our third aim was to investigate modification of respiratory TB's effect on airway disease. We hypothesized a higher risk of airway disease among TB patients. In terms of effect measure modification, we hypothesized higher relative risk for airway disease by TB in younger age groups [6], females [17], people born in countries with higher TB incidence [6], people who were not classified as ‘at personal health risk’ by our proxy variable [18], people without baseline Charlson comorbidities [6], and people in higher socioeconomic classes (due to expected lower exposure to airway disease risk factors).

## 2 Methods

### 2.1 Study design and participants

We conducted a retrospective cohort study based on linked immigration, public health surveillance, and health administrative data to estimate the absolute and relative risk of incident airway disease and death among people immigrating to BC who completed treatment for respiratory TB, compared with non-TB controls. Population-wide health administrative data were sourced from the Province of BC, Canada, and the Government of Canada, with access provided through Population Data BC (Supplementary Methods) [19]. CAB and MEK accessed the data for this study's statistical analyses during March 2020-January 2021.

**Fig. 1:**
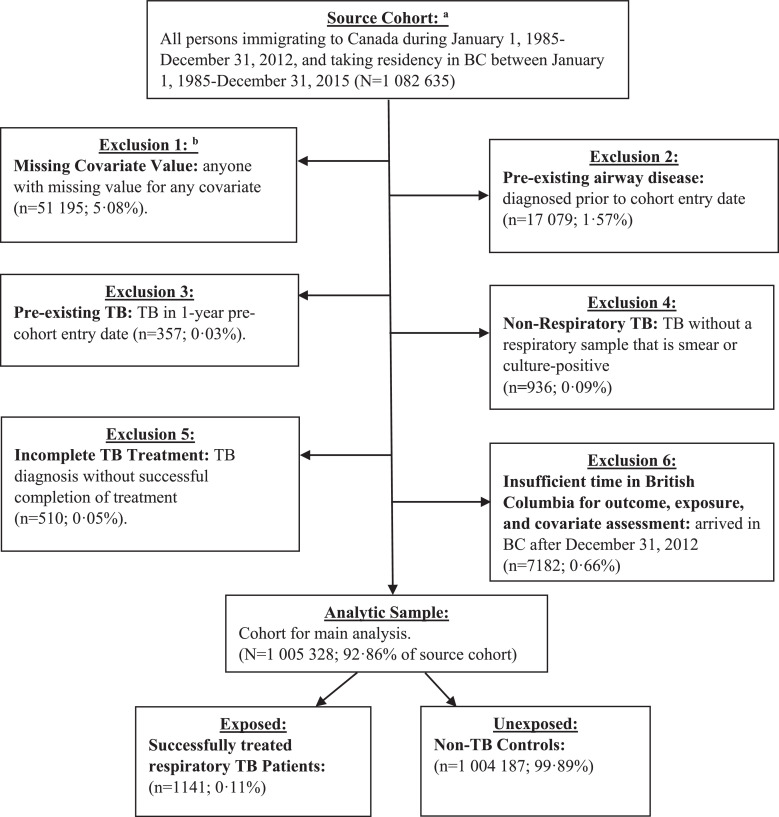
flowchart: analytic sample for post-tuberculosis airway disease among people immigrating to British Columbia, Canada, 1985–2015. Legend: BC = British Columbia, BCCDC = British Columbia center for Disease Control, IRCC = Immigration, Refugees, and Citizenship Canada, TB = tuberculosis. Notes: ^a^Ascertained from IRCC permanent resident database. Residency in BC defined by acquisition of provincial health insurance (MSP) registration minus 90 days, or first healthcare contact, whichever occurred first. ^b^Covariates for which people with a missing value were excluded were age, sex, income quintile, country of birth, immigration class, educational qualification, Charlson comorbidity score, or index year. Most people excluded in exclusion 3 were due to missing values for income quintile.

The source cohort included all persons immigrating to Canada and taking residency in BC during January 1st, 1985-December, 31st, 2015. The cohort entry date (CED) was set to 365 days after the date of residency in BC, which was defined as 90 days prior to provincial health insurance coverage date, or date of first healthcare contact (Supplementary Methods) [20,21]. The 365 days prior to CED was used as a covariate assessment window, and time after CED was used for exposure and outcome assessment [21]. We excluded from the analytic sample people with: missing covariate values, airway disease prior to CED, respiratory TB prior to CED, non-respiratory TB, not classified as “completed-successfully” in TB registry, or residence in BC after December 31, 2012 (Fig. 1). Altogether, these exclusion criteria removed 7.14% of participants (*n* = 77 307) from the source cohort.

Ethics approval was provided by the University of British Columbia (H16-00265). Patient consent is not required when data collected during the administration of healthcare are anonymized and used for research. We obtained approval from each data steward prior to accessing the data, and for the manuscript.

### 2.2 Outcome: airway disease

The primary outcome for our study, time-to-airway disease, was defined by total respiratory morbidity (TRM), which is an indicator variable developed by the Manitoba Centre for Health Policy for use with health administrative data in order to overcome coding variability between providers, patients of differing ages, and over time [22]. First published in 1993 [23], TRM has been used in various publications, spanning almost three decades, as a measure of airway disease incidence and prevalence in administrative data [22]. TRM was measured by the following diagnostic codes (ICD-9-CM: 466, 490–493, and 496; ICD-10-CA: J20-J21, J40-J45): asthma, bronchitis, bronchiolitis, emphysema, and chronic airway obstruction [22]. In our cohort, airway disease was ascertained from BC's hospital discharge abstracts, outpatient physician claims, and vital statistics death certificates. To meet our definition of airway disease, we required a single hospital visit (any diagnosis field), three or more physician visits within a 1-year period, or a death certificate with one of the codes above as the primary cause. The first date of hospital admission, physician service, or death with airway disease, was considered the event date for survival analysis. Participants were censored if they did not meet the airway disease definition prior to: death from another cause, loss of provincial health insurance coverage (proxy for residence in BC), or December 31, 2015 (end of study period). Time-to-airway disease, the outcome variable for our survival analyses, was calculated in person-years from the cohort entry date to censoring/event date.

### 2.3 Exposure: respiratory tuberculosis

Our exposure variable was defined microbiologically confirmed respiratory TB, which was measured using BC center for Disease Control (BCCDC) TB registry data. In this definition, people with confirmed TB with a respiratory sample (sputum = 71·4%, bronchial washings = 10·3%, pleural fluids = 4.8%, other = 13.5%) from a respiratory system site (lung = 84.5%, bronchi = 9.9%, pleura = 3.3%, other = 2.3%) that tested positive for TB on acid-fast bacilli (AFB) smear (52·3%) or culture (47·7%) were considered exposed persons (*n* = 1141). People diagnosed with non-respiratory TB were excluded. People without successful completion of TB treatment were also excluded from the analytic sample in order to provide a more conservative estimate of respiratory TB's effect on the risk of airway disease [14,24,25]. Successful treatment completion was defined using the treatment outcome description (value: “complete-successful”) registered in the BCCDC TB registry's treatment dataset, which is maintained by TB surveillance staff, in collaboration with TB clinic staff, according to Canadian TB Standards [26].

### 2.4 Covariates

**Fig. 2:**
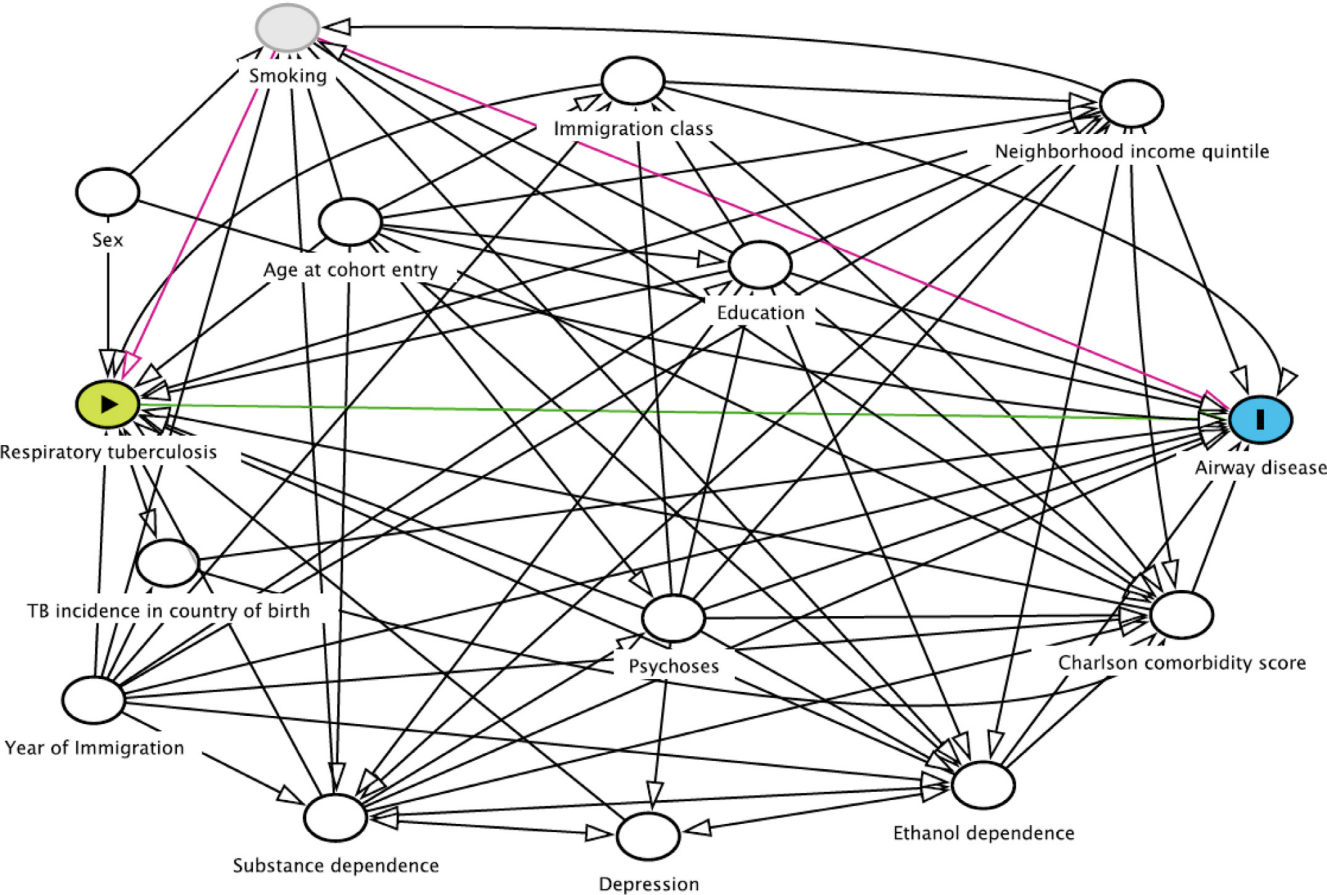
Directed acyclic graph (DAG) for post-tuberculosis airway disease among people immigrating to British Columbia, Canada, 1985–2015. Legend: TB = tuberculosis. Notes: Developed using Daggity online tool. Respiratory tuberculosis is defined from TB registry data. Airway disease includes chronic airway obstruction, asthma, bronchitis, bronchiolitis, and emphysema.

Our directed acyclic graph (DAG) identifies assumed interrelationships among baseline covariates, respiratory TB, and airway disease (Fig. 2). Baseline covariates were defined in a 1-year covariate assessment window prior to CED (Supplementary Figure S1). Age at CED, sex, income quintile of neighbourhood at CED [6,27], educational qualification (less than secondary, secondary, college or trades school, university) at CED [6,7,27], weighted Charlson comorbidity score (continuous) prior to CED [28], TB incidence in birth country (<100, 100–200, and 300+ cases per 100,000 annually) at CED [6], and year of residency in BC (to account for declining TB prevalence and rising airway disease prevalence over the study period) [29,30]. Ethanol dependence (ETOH), drug abuse, depression, and psychoses are associated with both TB and airway disease [31,32], and were adjusted for in the analysis ascertained from physician claims or hospital visits and Elixhauser comorbidity index definitions [28].

### 2.5 Statistical analysis

We first calculated airway disease incidence rates among exposed (TB) and unexposed (non-TB controls) to understand the absolute risk of airway disease during follow-up. We then plotted a Kaplan-Meier curve to assess airway disease-free survival between respiratory TB patients and controls, as well as the cumulative incidence of airway disease in each group, during follow-up. Data management was conducted in SAS 9.4 while statistical analyses were conducted in R v. 3.6.1.

#### 2.5.1 Aim 1: analyzing post-tuberculosis airway disease risk

Cox proportional hazards (PH) regression was used for our analyses of time-to-airway disease, with the adjusted hazard ratio (aHR) as our measure of association. First, we conducted univariable Cox PH regressions of time-to-airway disease on each covariate. Second, we conducted an age and sex-adjusted analysis of time-to-airway disease by TB status. Third, we included, as main effects, all variables identified in the DAG as potential confounders in a multivariable Cox PH regression of time-to-airway disease (main analysis). Sensitivity analyses included: (i) specifying a reduced Cox PH model that excluded ETOH, substance dependence, psychoses, and depression variables, which had relatively low numbers; (ii) replacing the weighted Charlson comorbidity score with the van Walraven-weighted Elixhauser comorbidity score [33], to check for consistency in the effect estimate; (iii) adding bronchiectasis and fibrosis codes (ICD9=494, 515, and ICD10=J47, J84) to the airway disease definition used in the main analysis; and (iv) removing TB patients with pleural samples (*n* = 55) from the exposure group. Additional sensitivity analyses changed the definition of TB used in the study: we first analyzed all forms of TB; second, we analyzed people with non-respiratory TB; last, we analyzed airway disease among people with pleural TB vs people with non-pleural respiratory TB.

#### 2.5.2 Aim 2: assessing potential unmeasured confounding

We conducted several sensitivity analyses to address the potential for unmeasured confounding. For example, smoking is a well-known risk factor common to TB and airway disease [6,34], but this variable is not generally available in health administrative databases, including ours. To examine the theoretical ability for an unmeasured confounder to explain away our effect estimate, we calculated E-values [35,36], based on the prevalence of the outcome in our study population and our main analysis results. An E-value tell us what the minimum adjusted association an unmeasured confounder would have to have with both the exposure variable and the outcome variable to nullify the effect estimate of interest [35]. To assess the robustness of the primary analysis’ effect estimates, we took the following approaches: (i) a propensity scores (PS) approach that included the covariates used in the primary analysis in a logistic regression PS model and then adjusted for deciles of the PS in a Cox PH regression outcome model; (ii) we incorporated empirical covariates (i.e., proxy variables from added data dimensions) identified through the hdPS algorithm (Supplementary Methods), in addition to the covariates used in the primary analysis, as additional covariate adjustment [37]; (iii) we used a hybrid of the hdPS algorithm and the least absolute shrinkage and selection operator(LASSO), to eliminate highly collinear proxy variables from the hdPS model above while keeping the primary analysis covariates (Supplementary Methods) [38]; (iv) we used a subset of the analytic sample that were listed in the BCCDC TB Registry's “Person Table” that had a tobacco use variable (yes/no) available; and (v) we created a “personal health risk” proxy variable for smoking behavior from the tobacco use variable in the BCCDC TB Registry, which was available for <5% of the cohort, and supplemented it with administrative data available for the entire cohort. This personal health risk proxy variable was included as an additional adjustment variable added to the main analysis. Administrative codes in physician claims and hospital abstracts were used to ascertain ‘personal health risk’, including codes related to: respiratory symptoms, cardiac symptoms, harmful exposures, personal health histories, substance dependence, nutrition and weight issues, and pregnancy complications (Supplementary Table S1). In addition to TB Registry tobacco use data, physician claims data, and hospital abstracts data, pharmacy dispensations data for bupropion, varenicline, or nicotine replacement therapies were added to the algorithm for ascertaining baseline personal health risk (Supplementary Methods).

#### 2.5.3 Aim 3: investigating effect measure modification

We created a series of separate adjusted Cox PH regression models to assess modification of the effect of respiratory TB on airway disease by: age group (age <40 vs 40+ years) [6], sex, TB incidence in country of birth (<200 vs 200+ cases annually per 100,000 population) [6], neighborhood income quintile, personal health risk, education level, immigration class, baseline weighted Charlson comorbidity score (0, 1, 2+), and depression. In these analyses, we used Cox PH regressions with a variable for respiratory TB, in addition to the main analysis covariates, a covariate for the hypothesized effect modifier, and an interaction term for respiratory TB by the hypothesized effect modifier. Each effect modification term was analyzed separately.

### 2.6 Role of the funding source

The funding sources had no role in this manuscript.

## 3 Results

### 3.1 Cohort characteristics

There were 1 005 328 people in our analytic sample (Fig. 1) contributing 11 202 533 person-years of follow-up. The percentage of respiratory TB patients in our cohort was 0·11% (*n* = 1141). Supplementary Table S2 presents the first diagnosis code for people who developed airway disease stratified by respiratory TB status. The median follow-up time for respiratory TB patients was 11·83 years (inter-quartile range (IQR) = 6·25–18·91), and 9·75 years (IQR = 5·08–16·75) for non-TB-diagnosed controls. During follow-up, 42·5% (*n* = 485) of respiratory TB patients developed airway disease after successful treatment completion, compared to 11·6% (*n* = 116 355) of non-TB controls (*p*<0·0001, Supplementary Table S3). In participants developing airway disease (median follow-up = 6·60 years, IQR = 3·03–11·78), compared to those censored (median follow-up = 10·25 years, IQR = 5·49–17·41), we identified a higher proportion of: TB patients, females, older aged, lower neighbourhood income, lower education, non-economic immigration classifications, earlier date of immigration to BC, higher mean Charlson comorbidity score, ETOH, substance dependence, psychoses, depression, and people classified as ‘at personal health risk’ by our proxy variable (Table 1). Univariable analyses indicated that all presumed confounders were associated with the outcome.

**Table 1:** Cohort characteristics stratified by outcome (airway disease or censored) among people immigrating to British Columbia, Canada, 1985–2015.

Characteristic	Censored N (%)	Airway disease N (%)	Crude HR	95% CI
Total	888 488	116 840		
Respiratory tuberculosis	656 (0·07)	485 (0·42)	3·24*	2·96–3·54
Follow-up time (mean (SD))	11·56 (7·36)	7·97 (6·02)		
Sex				
Female	455 737 (51·29)	62 248 (53·28)	Ref	Ref
Male	432 751 (48·71)	54 592 (46·72)	0·95*	0·94–0·96
Age, years (mean (SD))	32·41 (16·33)	35·04 (18·81)	1·01*	1·01–1·01
Neighbourhood income quintile				
Highest 20%	133 301 (15·00)	13 468 (11·53)	Ref	Ref
Middle-High 20%	128 939 (14·51)	15 001 (12·84)	1·14*	1·12–1·17
Middle 20%	161 746 (18·20)	21 343 (18·27)	1·28*	1·25–1·31
Low-Middle 20%	201 033 (22·63)	29 692 (25·41)	1·40*	1·37–1·43
Lowest 20%	263 469 (29·65)	37 336 (31·95)	1·37*	1·35–1·40
Education level				
None/Unknown	109 282 (12·30)	18 657 (15·97)	Ref	Ref
Secondary or less	374 619 (42·16)	57 554 (49·26)	0·76*	0·74–0·77
Trade/diploma	164 367 (18·50)	20 595 (17·63)	0·65*	0·64–0·66
University degree	240 220 (27·04)	20 034 (17·15)	0·54*	0·53–0·55
Immigration class				
Economic	544 098 (61·24)	50 362 (43·10)	Ref	Ref
Family	253 158 (28·49)	50 522 (43·24)	1·86*	1·84–1·88
Refugee	23 900 (2·69)	3957 (3·39)	1·52*	1·49–1·55
Other	67 332 (7·58)	11 999 (10·27)	1·49*	1·44–1·54
TB incidence rate in country of origin at time of immigration				
<100 per 100 000 pop·	381 530 (42·94)	36 967 (31·64)	Ref	Ref
100 to <200 per 100 000 pop·	292 116 (32·88)	35 552 (30·43)	1·02*	1·00–1·03
200 to <300 per 100 000 pop·	112 878 (12·70)	30 077 (25·74)	2·01*	1·98–2·04
300+ per 100 000 pop·	101 964 (11·48)	14 244 (12·19)	1·23*	1·20–1·25
Year of immigration (mean (SD))	15·16 (7·01)	10·21 (6·16)	0·96*	0·96–0·96
Charlson comorbidity score (mean (SD))	0·05 (0·33)	0.09 (0·37)	1·38*	1·37–1·39
Ethanol dependence				
No	888 331 (99·98)	116 442 (99·70)	Ref	Ref
Yes	157 (0·018)	398 (0·30)	2·02*	1·83–2·23
Substance dependence				
No	888 359	116 440 (99·70)	Ref	Ref
Yes	129 (0·015)	400 (0·30)	1·75*	1·59–1·93
Psychosis				
No	888 314 (99·98)	116 366 (99·60)	Ref	Ref
Yes	174 (0·02)	474 (0·40)	1·53*	1·40–1·68
Depression				
No	883 806 (99·47)	107 651 (92·10)	Ref	Ref
Yes	4682 (0·53)	9189 (7·90)	1·78*	1·75–1·82
Personal health risk proxy variable				
No	826 181 (92·99)	101 531 (86·90)	Ref	Ref
Yes	62 307 (7·01)	15 309 (13·10)	1·83*	1·80–1·86

**Legend:** CI = confidence interval; ETOH = ethanol dependence; HR =hazard ratio; Ref = reference category for HR; asterisk (*) indicates result was significant at α=0·05.  **Notes:** The censored group includes all people who did not experience the event (airway disease) prior to or at the time of death, leaving BC, or study end date (December 31, 2015).  Univariable Cox proportional hazards regressions estimated hazard ratios for airway disease by each covariate.

### 3.2 Statistical analysis results

**Fig. 3:**
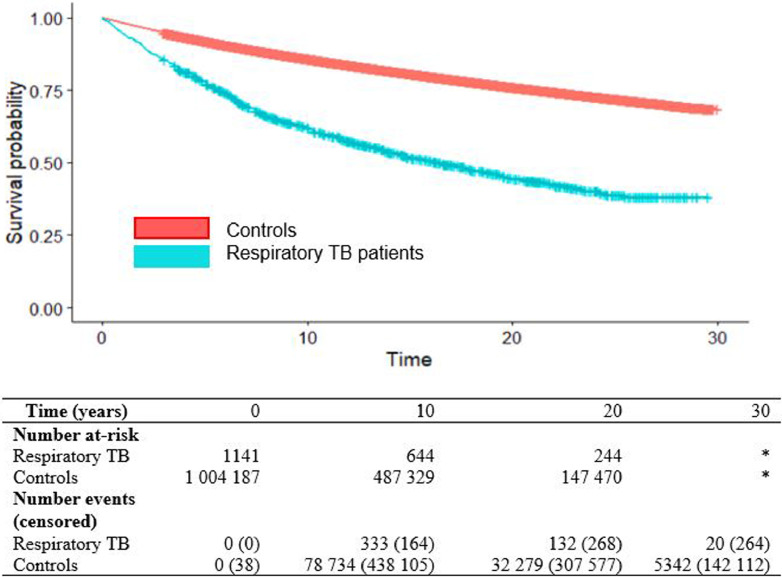
Kaplan–Meier plot for airway disease-free time (years) among people immigrating to British Columbia, Canada 1985–2015: respiratory tuberculosis patients vs non-tuberculosis controls. Legend: teal = respiratory TB; red = non-TB controls. Notes: Asterisk (*) indicates suppressed due to cell count <6, or cross-suppressed as next lowest value.

The Kaplan-Meier curve showed a large the difference in airway disease-free survival between people with and without respiratory TB (Fig. 3). In terms of absolute risk, we found that airway disease was diagnosed in 116 840 people during follow-up, yielding an incidence rate of 10·29 per 1000 person-years, which differed between respiratory TB patients and non-TB controls (33·73 vs 10·04, respectively). In terms of relative risk, our main analysis found a covariate-adjusted hazard ratio of 2·08 (95% CI: 1·91–2·28) times the risk of airway disease among people diagnosed and completing treatment for airway disease compared with non-TB controls (Table 2). A sensitivity analysis that removed ETOH, drug dependence, psychoses, and depression variables had a similar result to the main analysis (aHR=2·11, 95% CI: 1·93–2·30). Replacing the Charlson comorbidity score with the van Walraven-weighted Elixhauser score changed the aHR to 2·06 (95% CI: 1·89–2·26). When bronchiectasis and fibrosis codes were added to the outcome definition, the adjusted HR increased to 2·18 (95% CI: 2·00–2·18). When TB patients with pleural samples (*n* = 55) were removed from the exposure group, the updated effect of TB was aHR=2·10 (95% CI: 1·92–2·30). People with any type of TB had a 75% increased risk of airway disease, while people with non-respiratory TB experienced a 36% increased risk of airway disease (Table 2). Among people with respiratory TB, there was no significant difference between those with pleural vs non-pleural TB (aHR=0·87; 95% CI: 0·57–1·32; Table 2).

**Table 2:** Cox proportional hazards regression analyses: time-to-airway disease among people immigrating to British Columbia, Canada, 1985–2015.

Statistical Analysis^a^	N	Adjusted HR	95% CI
*Aim 1: analyzing post-TB airway disease risk*			
Covariate-adjusted (main analysis: respiratory TB vs controls)	1 005 328	2·08	1·91 – 2·28
Sensitivity analyses			
Covariate-adjusted (removed ETOH, substance dependence, psychoses, and depression)^b^	1 005 328	2·11	1·93 – 2·30
Covariate-adjusted (van Walraven-weighted Elixhauser comorbidity score)^c^	1 005 328	2·06	1·89 – 2·26
Covariate-adjusted (bronchiectasis and fibrosis added to the airway disease definition)	1 005 283	2·18	2·00 – 2·38
Covariate-adjusted (removed respiratory TB patients with pleural samples; *n* = 55)	1 005 273	2·10	1·92 – 2·30
*Different TB definitions*			
Covariate-adjusted (all forms of TB vs controls)	1 006 271	1·75	1·63 – 1·88
Covariate-adjusted (non-respiratory TB vs controls)^d^	1 004 733	1·36	1·20 – 1·53
Age/sex-adjusted (pleural TB vs non-pleural TB)	1141	0·87	0·57 – 1·32
*Aim 2: assessing potential unmeasured confounding*			
*PS methods*			
PS decile-adjusted (main covariates)	1 005 328	2·27	2·08 – 2·49
hdPS decile-adjusted (main covariates + empirical covariates)	1 005 328	2·28	2·09 – 2·50
LASSO-hdPS decile-adjusted (main covariates + LASSO-refined empirical covariates)	1 005 328	2·26	2·07 – 2·47
*Adjustment for smoking behavior proxy variables*			
Covariate-adjusted subdata analysis (main covariates + tobacco use variable)^e^	31 063	1·53	1·37 – 1·71
Covariate-adjusted (main covariates + personal health risk proxy variable)	1 005 328	2·03	1·85 – 2·22

BC = British Columbia; BCCDC = British Columbia center for Disease Control; CI = confidence interval; ETOH = ethanol dependence; hdPS = high-dimensional propensity score; HR = hazard ratio; LASSO = least absolute shrinkage and selection operator; *N* = analytic sample size; PS = propensity score; TB = tuberculosis.

Notes:

^a^Covariate-adjusted models included as adjustment variables: age at index, sex, income quintile at index, educational qualification upon immigration, immigration class, TB incidence in country of birth, weighted Charlson comorbidity score, year of arrival in BC, ETOH, substance dependence, psychoses, and depression.

^b^Due to lower than expected numbers, we removed these three variables from the set of adjustment variables.

^c^We replaced the Charlson comorbidity score with the van Walraven-weighted Elixhauser comorbidity score and removed ETOH, substance dependence, psychoses, and depression variables as individual binary covariates because they are included in the van Walraven Elixhauser comorbidity score.

^d^Non-respiratory TB included everyone who was a confirmed case of TB but did not have a smear or culture positive respiratory sample in the laboratory datasset of the BCCDC TB Registry.

^e^This analysis used a lower number of cohort members because it includes only people who were in the BCCDC TB Registry's Person dataset, which is a client list used by Provincial TB Services to track people who are tested or treated for active or latent TB within BC.

In our assessment of potential unmeasured confounding by smoking, we calculated an E-value of 3·58 for the main analysis’ aHR, and an E-value of 3·23 for the 95% confidence limit closest to the null. A standard PS analysis estimated an aHR of 2·27 (95% CI: 2·08–2·49) for airway disease by respiratory TB, while PS analyses utilizing additional data dimensions for proxy adjustment of unmeasured confounding via hdPS empirical covariates yielded aHR's ranging from 2·26 to 2·28 (Table 2). A subgroup analysis of the cohort with non-missing values for tobacco use (*n* = 31 063; 3·1% of analytic sample) estimated an aHR=1·53 (95% CI: 1·37–1·71). A sensitivity analysis that added an investigator-defined proxy variable for “personal health risk” showed slight attenuation of the main analysis result (aHR=2·03, 95% CI: 1·85–2·22).

There was no discernible pattern of effect measure modification by age group, sex, education level, income quintile, immigration class, or by our personal health risk variable (Supplementary Table S4). We observed a higher aHR for airway disease by respiratory TB among people born in lower-TB-incidence countries with aHR=2·57 for people from countries with <200 TB cases annually per 100,000 population, compared with aHR=1·79 for people born in countries with 200+ TB cases annually per 100,000 population (Supplementary Table S4). Effect measure modification was also observed in the relationship between TB and airway by comorbidity level, with aHR=2·25 among people with no Charlson comorbidities at baseline, compared with people with 1 or 2+ Charlson comorbidities at baseline (aHR=1·04 and aHR=1·52, respectively).

## 4 Discussion

In a cohort of people immigrating to BC, Canada, from 1985-2015, 42·5% of people diagnosed with respiratory TB developed airway disease compared to 11·6% of non-TB controls (rates were, respectively: 33·73 and 10·04, per 1000 person-years). After covariate adjustment, we found a 108% increased risk of airway disease in cohort members diagnosed with respiratory TB than non-TB controls. This finding was robust to multiple sensitivity analyses. In aim 2, we attempted to assess the impact of potential unmeasured confounding through multiple sensitivity analyses, which further supported the main analysis conclusion. In aim 3, we found evidence of effect measure modification of the effect of TB on airway disease risk by level of comorbidity and by TB incidence rate in country of birth.

Our finding of increased risk of airway disease, post-TB treatment completion, was consistent with existing systematic reviews, which concluded that people diagnosed with TB are at increased risk of various forms of respiratory disease [5], [6], [7], [8]. The most recent published meta-analysis, by Byrne et al. [6], found a pooled odds ratio (pooled OR) of 3·05 (95% CI: 2·42–3·85) for chronic respiratory disease among people previously treated for TB compared to people without a history of TB. However, in that meta-analysis, only one study was a cohort study, which reported an adjusted HR of 2·05 (95% CI: 1·77–2·39) [39], and was closer to our main analysis estimate than other estimates in that meta-analysis [6].

Two studies of post-TB airway disease risk from high-resource low-TB-incidence settings were published at the time of writing, both observing increased risk of respiratory disease by active TB diagnosis: one from United States (case-control study) with aOR=5.37 (95% CI: 2.98–9.68) for pulmonary impairment by active TB vs latent TB patients [14], and another from Finland (cross-sectional study) with aOR=2.68 (95% CI: 2.00–3.61) for COPD by past TB vs no TB [13]. Both studies made efforts to control for potential confounders in their cross-sectional analyses. Pasinapodya et al. acknowledged the lack of ability to control for pre-existing lung impairment [14], while Mattila et al. were able to exclude people with asthma prior to study enrollment [13], leading their result closer to our main analysis result [13]. The limitations of these cross-sectional studies may imply an overestimation of post-TB airway disease risk compared with our study, although differences in populations, study designs, and measures of association limit comparability. Numerous other studies from higher TB-incidence settings have consistent findings with ours [18,[39], [40], [41], [42]].

In terms of effect modification, our findings do not align with those of Byrne et al., who noted increasing study log-odds ratios for post-TB airway disease by increasing national TB incidence rate for the country in which the study was conducted [6]. Byrne et al. also suggested effect modification by age group, with younger age groups (<40 years) being at higher risk of COPD by TB history [6]. However, our analysis did not find significant effect measure modification by age. Importantly, difference in study design (cohort study vs meta-analysis) may explain the incongruent findings between our study and that of Byrne et al. [6]. The finding that people from lower-TB-incidence countries had higher aHR than people from higher-TB-incidence countries may indicate lower exposure to risk factors for airway disease among people from lower-TB-incidence countries. A lower level of exposure to airway disease risk factors may, in turn, have made TB a potentially greater impact in this group. This speculation is supported by a finding of lower mean Charlson comorbidity score and lower proportion at personal health risk at baseline in people from countries with TB incidence <200 per 100,000 annually. A similar phenomenon may explain why people with no baseline comorbidities experienced a greater effect of respiratory TB on airway disease risk. These effect modification findings, and our speculation about their meaning, warrant further investigation.

Ravimohan et al. recently reviewed the topic of post-TB airway disease and noted the lack of precise understanding of the pathophysiological mechanisms in generating post TB airway disease [7]. Ravimohan et al. recommended population-based epidemiological studies that examine the prevalence of various types of airway deficits stratified by key risk groups (e.g., HIV, diabetes) and suggest immunopathogenesis and genome-wide association studies for correlates of lung damage and hyper-inflammation. In clinical research, studies of diagnostics during and after TB treatment, and investigations of adjunctive host-directed therapies and common COPD medications, are recommended. Our study did not seek to differentiate between subtypes of airway disease, nor to explore mechanisms by which airway disease develops. However, we observed a higher proportion of emphysema, chronic airway obstruction, and chronic bronchitis among people diagnosed with respiratory TB than controls, which may be expanded upon in future studies. When bronchiectasis and fibrosis were added to our definition of airway disease, we observed an increase in the aHR from 2.08 to 2.18, suggesting additional disease burden when more forms of respiratory disease are considered.

Our study overcomes the temporality limitation of previous cross-sectional studies [6], [7]. We used a large (>1 M) population-based longitudinal cohort containing data for all people immigrating to BC over 30 years [20]. We adjusted for multiple known confounders and removed pre-existing TB and airway disease. Additionally, these population-based data enabled near-complete capture of physician visits, hospital encounters, and deaths related to the outcome within the province of British Columbia. We used legislated, population-based TB surveillance and laboratory data to ascertain exposure status and timing, which improves upon previous studies [6,7]. An advantage of our definition of airway disease (compared with individual ICD codes) is that, by including a family of diagnosis codes, this definition overcomes potential misclassification in longitudinal healthcare claims data due to code shifts over time, and is also robust to variations in coding practices between providers [22].

Our study is limited to the population of people immigrating to BC (a low-TB-incidence setting with high-income and universal public healthcare), and our results should not be generalized to domestic TB patients within similar settings, nor to higher TB-burden settings. As a study of healthcare claims data, these results are based on treatment data, and do not include symptomatic yet untreated airway disease. Like all observational studies, causality cannot be inferred from these data. We cannot rule out time-dependent confounding as the covariates used in our study were measured at baseline, which means changes over time in, for example, education or comorbidities, would not be registered.

Smoking was a known unmeasured confounder in our study. Based on our E-value calculation, an aHR≥3·58 for such an unmeasured confounder's associations with both TB and airway disease, adjusted for the covariates included in the main analysis, could explain away our main analysis result, but a weaker confounder could not. Moreover, an unmeasured confounder would need an adjusted association ≥3·23, on risk ratio scale, with both the exposure and the outcome, to render our main analysis result non-significant at α=0·05. We do not expect that such a strong independent confounder (specifically, smoking) was omitted from our analyses, based on an adjusted OR of 1·80 for “ever smoking” in a US case-control study of post-TB lung impairment [14]. However, E-values, are theoretical and not based on real data, they make the assumption of a symmetrical relationship of the unmeasured confounder with both exposure and outcome (generally unrealistic), and are subject to any biases already incorporated in the effect estimate (selection bias, reporting bias, etc.) [43].

Our exclusion of people with pre-existing airway disease from the cohort would have also removed a reasonable proportion of smokers at baseline (11·5% of excluded persons were classified as at “personal health risk” by our proxy variable, whereas, in the analytic sample, only 7·7% were classified as such). Adjustment for empirical covariates derived from the hdPS algorithm, deployed on hospital and physician claims data in sensitivity analyses, attempted to address unmeasured or residual confounding, yet showed a slight increase in the effect estimate for airway disease by respiratory TB status, compared with the standard PS model. PS approaches are not preferred in settings where exposure prevalence is rare (<1% in our case) [44], which is why regression was used in our main analysis, and may explain the difference between our main analysis aHR and aHRs from the PS analyses. Our subgroup analysis of participants in the TB registry, adjusted for tobacco use variable available for <5% of participants, as well as an analysis using a “personal health risk” proxy variable defined from administrative data available for the entire cohort, both found elevated risk of airway disease among people diagnosed with TB, reinforcing our main analysis. Irrespective of these analyses, smoking remains a known confounder that was unmeasurable in our study.

This study demonstrates that post-TB airway disease is a problem for TB survivors in low-TB-incidence, high-resource settings. There is a paucity of guidance on diagnosis and treatment of post-TB airway disease in high-resource regions [10,15], despite the widespread availability of pulmonary specialists, pulmonary function testing, and potential therapies such as pulmonary rehabilitation and pharmacologic measures. While unmeasured confounding by smoking is unlikely to explain our finding of increased airway disease risk among respiratory TB patients, TB programs should routinely collect and record information about smoking behavior and consider diagnosis with TB a teachable moment to promote smoking cessation. Achieving a fourth 90, “[e]nsuring that 90% of all people successfully completing treatment for TB can have a good health-related quality of life” [4], requires addressing post-TB airway disease [16]. Moreover, post-TB airway disease has a substantial health economic impact in terms of DALY estimates [45], in which acute-illness and mortality-based methods for assessing TB's impact accounted for only a quarter of estimated DALYs when chronic lung impairment was incorporated in DALY calculation in a low-TB-incidence setting [46]. Given our findings, along with data showing similar associations from other high-resource settings [13,14,47], we strongly urge investment in the diagnosis of post-TB airway disease, and research on potential evidence-based interventions [25], as well as a health economic re-evaluation of latent TB screening and treatment, in low-TB-incidence settings.

## Declaration of Competing Interest

CAB reports grants from Canadian Institutes of Health Research, during the conduct of the study. JCJ reports grants from Canadian Institutes for Health Research, and a grant from Michael Smith Foundation for Health Research, during the conduct of the study. MEK reports consultation fees from Biogen, Inc (unrelated to this content). All other authors declare no competing interests.

## Appendix Supplementary materials

Image, application 1
