# Supplementary material for: Post-tuberculosis airway disease: A population-based cohort study of people immigrating to British Columbia, Canada, 1985–2015
Source: eClinicalMedicine. 2021 Feb 26;33:100752. doi: 10.1016/j.eclinm.2021.100752 (PMC7933261; doi:10.1016/j.eclinm.2021.100752)
Supplement: Supplementary file 1 [file mmc1.docx]

**Supplementary Appendix**

**Post-tuberculosis airway disease: a population-based cohort study of people immigrating to British Columbia, Canada, 1985-2015**

Basham CA^1,2^, Karim ME^1,3^, Cook VJ^2,4^, Patrick DM^1,2,4^, Johnston JC^1,2,4^

1. School of Population and Public Health, University of British Columbia, Vancouver, Canada.

2. British Columbia Centre for Disease Control, Vancouver, Canada.

3. Centre for Health Evaluative and Outcome Sciences, University of British Columbia, Vancouver, Canada.

4. Department of Medicine, University of British Columbia, Vancouver, Canada.

**Contents:**

1. Supplementary methods text
   1. Health administrative data sources
   2. Assessment of unmeasured confounding using added data dimensions:
      1. High-dimensional propensity score method
      2. LASSO-hdPS method
   3. Post-TB airway disease among respiratory TB patients
   4. References
2. Supplementary figures
   - Figure S1: Design of retrospective cohort study of post-tuberculosis airway disease among immigrants to British Columbia, Canada, 1985-2015.
   - Figure S2. Kaplan-Meier plot of airway disease-free survival time (years) from TB diagnosis date among immigrants to British Columbia, Canada, 1985-2015 who were diagnosed with TB.
3. Supplementary tables
   - Table S1. Personal health risk proxy variable International Classification of Diseases (ICD) and physician billing claims codes from physician visits and hospital encounters.
   - Table S2: First diagnosis for people developing airway disease during follow-up among immigrants to British Columbia, Canada, 1985-2015: stratified by exposure group (respiratory TB vs controls).
   - Table S3. Cohort characteristics stratified by respiratory tuberculosis status among people immigrating to British Columbia, Canada, 1985-2015.
   - Table S4. Effect measure modification of airway disease risk by respiratory tuberculosis, modified by key covariates among people immigrating to British Columbia, Canada, 1985-2015.
   - Table S5. Cohort characteristics of people diagnosed with respiratory tuberculosis, stratified by airway disease or censoring (outcome) status among people immigrating to British Columbia, Canada, 1985-2015
4. **SUPPLEMENTARY METHODS TEXT**
5. **Health administrative data sources**

Individually linked, population-wide, health administrative data for people immigrating to British Columbia (BC), Canada, 1985-2015 were sourced from the Province of BC, and the Government of Canada, with access provided through Population Data BC.^1–8^ These data have been described elsewhere.^9–11^

1. **Assessment of unmeasured confounding using added data dimensions**

We attempted to apply the modified disjunctive cause criterion^12^ in our study by including empirical proxy covariates for potential unmeasured confounders, in addition to covariates selected using substantive knowledge of associations with the exposure and the outcome. Our study attempted to control for confounding using multiple methods because the performance of various methods in reducing potential bias in the association of interest in our study (respiratory TB and airway disease) is unclear. Below we describe the two methods employing added data dimensions for proxy adjustment of unmeasured or poorly measured confounders.

*(i) High-dimensional propensity score method*

The high-dimensional propensity score (hdPS) algorithm identified proxy variables, known as empirical covariates, from data dimensions (physician claims data and hospital abstracts data in this study). For determining the most relevant proxy or empirical covariates helpful to reduced the bias due to unmeasured or poorly measured confounding, this algorithm examines the prevalence and potential for reducing bias (association with the exposure and the outcome, separately) using a modification of the Bross formula.^13,14^ The seven steps in the hdPS algorithm are as follows: (1) include added data dimensions from which the proxy or empirical variables will be ascertained, (2) identify candidate empirical covariates using the *n* most prevalent codes (e.g., ICD-9-CM) within the added data dimensions (default is n=200), (3) determine frequency of code occurrence for these empirical covariates via three dummy variables for each code within each cohort member as either once, sporadically, or frequently (cov_once_=1 when occurs once; cov_sporadic_=1 if occurs more than the median number of times across cohort members; cov_frequent_=1 when occurs for that person more than the 75^th^ percentile across cohort members), (4) rank empirical covariates in terms of potential for causing/reducing bias via the Bross formula for binary covariates (wherein the relative risk of the binary covariate is used in conjunction with that covariate’s prevalence in the unexposed and exposed groups), and then sort them in descending order (5) choose a prespecified number of empirical covariates (k=400 in this study) based on their Bross formula bias assessment rank, (6) include any investigator-specified covariates (e.g., age, sex, education, etc.) with the selected empirical proxy variables (i.e., these selected variables are known as hdPS variables) identified in step 5 in an exposure variable (respiratory TB in our study) prediction model (e.g., logistic regression) to estimate the hdPS, (7) use the resulting hdPS in an exposure-outcome model, such as by grouping cohort members into hdPS deciles and including the hdPS decile variable, along with the exposure variable (respiratory TB in our study), in a regression model (Cox proportional hazards regression in our study of time-to-airway disease/censoring) we then estimated the effect of respiratory TB.^13^

VanderWeele describes the pros and cons of the hdPS in comparison with forward and backward (p-value based) methods used commonly in epidemiological effect studies.^12^ One advantage of the hdPS over p-value-based methods is its assessment of each variables potential for reducing biasing by measuring association with both exposure and outcome, whereas p-value (or information criteria)-based methods only check association with the outcome.^12^ A disadvantage of the hdPS is the inability to ensure a minimum adjustment set guaranteed to control for bias if the initial adjustment set is known to suffice to control for confounding, because the hdPS algorithm models each individual covariate individually.^12^ Additionally, the hdPS alone may result in overfitting in the final PS model. A second approach to the hdPS was taken that added a regularization technique to select the final list of proxy variables for inclusions in the hdPS estimation model.

*(ii) LASSO-hdPS method*

As the hdPS-generated proxy or empirical covariates for addressing unmeasured confounders are assessed individually, some or many of them may be highly collinear, creating unstable PS estimates (e.g., lack of overlap in the PS between exposure and control groups). We, therefore, used a hybrid of hdPS and least absolute shrinkage and selection operator (LASSO), whereby (a) we obtain the final set of empirical covariates or hdPS variables, and (b) these hdPS variables are then entered into a LASSO logistic regression for a binary version of the outcome (airway disease: censored or event).^15,16^ In this hybrid LASSO-hdPS method, we sought to reduce the number of proxy covariates generated by the hdPS algorithm to avoid potential overfitting in the PS model, which can lead to poor overlap in the PS between the exposed (respiratory TB) and unexposed (non-TB control) groups. The LASSO method works by shrinking the value of standardized covariate coefficients for the proxy variables to zero when, after applying the penalty term lambda, the variable is non-significantly associated with the outcome variable (airway disease) after controlling for the other covariates, including the investigator-specified covariates, as well as the exposure variable (respiratory TB).^15,16^ The final set of LASSO-refined hdPS variables are selected from a LASSO logistic regression model using a lambda value that minimized the mean squared error (MSE), with optimal lambda value identified through a grid search across the 5-folds of the data.^15,16^ The resulting LASSO-selected proxy variables were then included with the investigator-specified covariates (e.g., age, sex, etc.) in a standard logistic regression to predict the exposure (respiratory TB) and generate a LASSO-hdPS (step 6 in the hdPS algorithm). These propensity scores are then used to create PS deciles that are adjusted for in the outcome Cox PH regression for time-to-airway disease by respiratory TB (step 7 in the hdPS algorithm).

1. **Post-TB airway disease among respiratory TB patients**

To compare airway disease among people diagnosed with respiratory TB, we created an outcome-stratified table of the covariates, with tests for differences between airway disease and censored groups by Chi-square tests for categorical variables and t-tests for continuous variables (Table S5).

**References**

1. BC Ministry of Health [creator]. Consolidation File (MSP Registration & Premium Billing). Population Data BC
2. [publisher]. Data Extract. MOH (2018). 2018. www.popdata.bc.ca/data.
3. Immigration Refugees and Citizenship Canada [creator]. Permanent Resident database: Population Data BC [publisher]. Data Extract. IRCC (2015). www.popdata.bc.ca/ data. Published 2015.
4. Canadian Institute for Health Information [creator]. Discharge Abstract Database (Hospital Separations). Population Data BC [publisher]. Data Extract. MOH (2018). 2018.
5. BC Ministry of Health [creator]. Medical Services Plan (MSP) Payment Information File. Population Data BC [publisher]. Data Extract. MOH (2018). 2018. www.popdata.bc.ca/data.
6. BC Vital Statistics Agency [creator]. Vital Statistics Deaths. V2. Population Data BC [publisher]. Data Extract BC Vital Statistics Agency (2018). 2018. www.popdata.bc.ca/data.
7. BC Centre for Disease Control [creator]: BC Provincial TB Registry (BCCDC-iPHIS). Population Data BC [publisher]. Data Extract. BCCDC (2018). Available: www.popdata.bc.ca/data (accessed 2018 Jan. 31).
8. BC Ministry of Health [creator]: PharmaNet. BC Ministry of Health [publisher]. Data Extract. Data Stewardship Committee (2018). Available: www. popdata.bc.ca/data (accessed 2018 Jan. 31).
9. Population Data BC. Data access. https://www.popdata.bc.ca/dataaccess. Published 2018. Accessed October 30, 2020.
10. Ronald LA, Campbell JR, Balshaw RF, et al. Predicting tuberculosis risk in the foreign-born population of British Columbia, Canada: study protocol for a retrospective population-based cohort study. BMJ Open. 2016;6(11):e013488.
11. Ronald LA, Campbell JR, Balshaw RF, et al. Demographic predictors of active tuberculosis in people migrating to British Columbia, Canada: a retrospective cohort study. Can Med Assoc J. 2018;190(8):E209-E216.
12. Vanderweele TJ. Principles of confounder selection. Eur J Epidemiol. 2019;34(3):211-219.
13. Schneeweiss S, Rassen JA, Glynn RJ, Avorn J, Mogun H, Brookhart MA. High-dimensional Propensity Score Adjustment in Studies of Treatment Effects Using Health Care Claims Data. Epidemiology. 2009;20(4):512-522.
14. Wyss R, Fireman B, Rassen JA, Schneeweiss S. Erratum: High-dimensional Propensity Score Adjustment in Studies of Treatment Effects Using Health Care Claims Data. Epidemiology. 2018;29(6):e63-e64.
15. Franklin JM, Eddings W, Glynn RJ, Schneeweiss S. Regularized regression versus the high-dimensional propensity score for confounding adjustment in secondary database analyses. Am J Epidemiol. 2015;182(7):651-659.
16. Karim ME, Pang M, Platt RW. Can We Train Machine Learning Methods to Outperform the High-dimensional Propensity Score Algorithm? Epidemiology. 2018;29(2):191-198.
17. **SUPPLEMENTARY FIGURES**

**Figure S1: Design of retrospective cohort study of post-tuberculosis airway disease among immigrants to British Columbia, Canada, 1985-2015.**


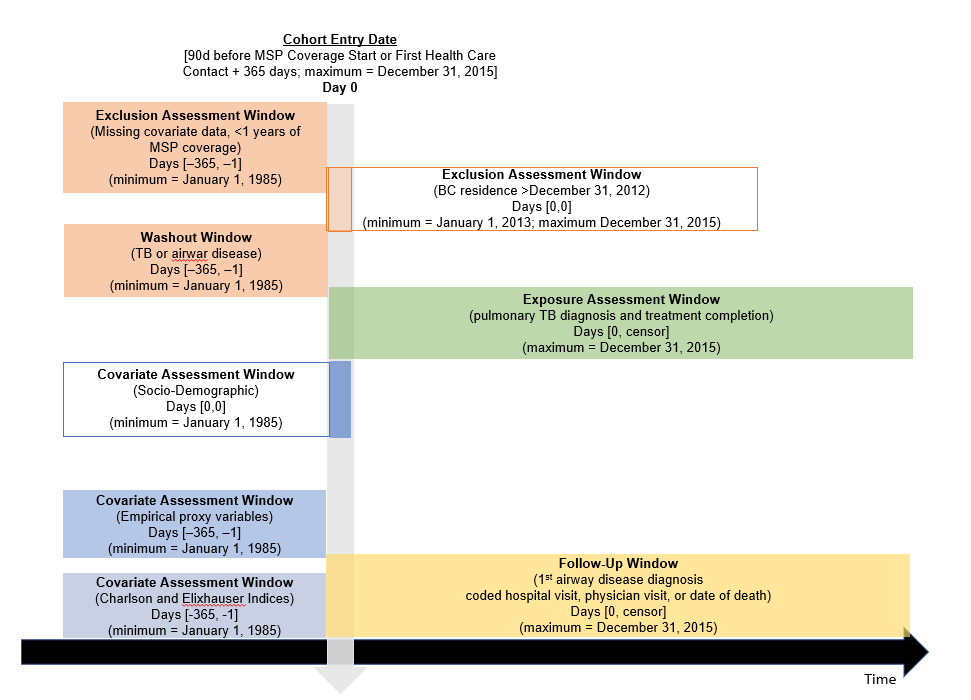


**Acronyms:** MSP, Medical Services Plan of British Columbia.

**Notes:** The MSP is a population-wide provincial health insurance registry that provides a reliable population-based denominator for the province of British Columbia.

**Figure S2. Kaplan-Meier plot of airway disease-free survival time (years) from TB diagnosis date among immigrants to British Columbia, Canada, 1985-2015 who were diagnosed with TB**


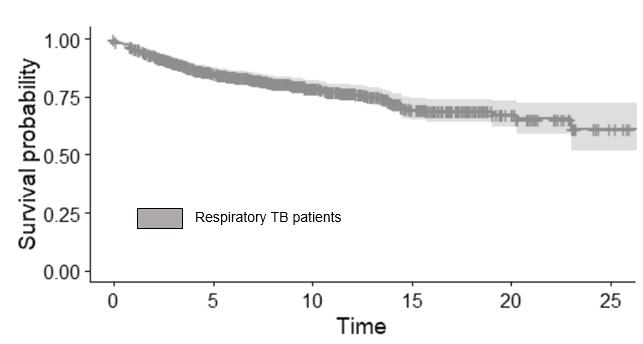


|  |  |  |  |  |  |  |
| --- | --- | --- | --- | --- | --- | --- |
| **Follow-up** | **0 years** | **5 years** | **10 years** | **15 years** | **20 years** | **25 years** |
| Number at-risk | 1141 | 526 | 282 | 119 | 39 | 6 |
| Number events (censored) | <5 (313) | 112 (187) | 30 (214) | 23 | <5 (77) | <5 (31) |

**Notes:** <5 = suppressed for privacy reasons.

1. **SUPPLEMENTARY TABLES**

**Table S1. International Classification of Diseases (ICD) and billing claims codes for physician visits and hospital encounters used to generate a proxy variable for personal health risk**

| **Variable component** | **BC MSP Fee Item** | **ICD-9** | **ICD-10** |
| --- | --- | --- | --- |
|  |  |  |  |
| Respiratory or chest symptoms | - | 786 | R069, R064, R0601, R0681, R063, R0602, R0682, R062, R0600, R0609, R063, R0683, R0689, R061, R05, R042, R049, R0481, R0489, R093, R079, R072, R071, R0781, R0782, R0789, R222, R0989, R066, R0689 |
| Cardiovascular symptoms | - | 785 | R000, R002, R011, R012, I96, R579, R570, R6521, R571, R578, R599, R0989, |
| Non-dependent abuse of drugs | - | 305 | F17200, F1010, F1210, F1290, F10610, F10310, F1110, F10610, F1410, F1510, F1910, F1810, F10610 |
| Nutrition, metabolism, and development | - | 783 | R630, R635, R634, R636, R633, R6250, R6251, R620, R6252, R631, R632, R627, R638, R635, |
| Other personal history presenting hazards to health | - | V15 | Z91010, Z91011, Z91012, Z91013, Z91018, Z91038, Z91040, Z91041, Z91048, Z9889, Z98870, Z98871, Z9889, Z923, Z91410, Z91411, Z91412, Z9149, Z8781, Z87820, Z87821, Z87828, Z9189, Z920, Z9283, Z9119, Z87891, Z283, Z77090, Z578, Z77011, Z9281, Z9181, Z779, Z9289, Z9189, |
| Toxic effect of other substances chiefly nonmedicinal as to source | - | 989 | T63, T55, T64, T65, T57 |
| Other conditions or status of the mother complicating pregnancy, childbirth, or the puerperium | - | 649 | O99, 026, 075 |
| Personal health risk assessment physician consultation | G14066 | - | - |

**Legend:** BC = British Columbia, Fee Item = MSP billing code, MSP = Medical Services Plan of British Columbia.

**Notes:** The first date of a physician visit, hospital encounter, or prescription dispensation was used to determine whether the proxy variable definition was met within the covariate assessment period, 1 to 365 days prior to cohort entry date.

**Table S2: First diagnosis for people developing airway disease during follow-up among immigrants to British Columbia, Canada, 1985-2015: stratified by exposure group (respiratory TB vs controls)**

| **Type of airway disease** | **Controls [*n* (%)]** | **Respiratory TB [*n* (%)]** |
| --- | --- | --- |
| Acute bronchitis | 57 043 (49·0) | 217 (44·7) |
| Asthma | 31 237 (26·8) | 95 (19·6) |
| Bronchitis | 19 961 (17·2) | 87 (17·9) |
| Chronic airway obstruction | 4491 (3·9) | 47 (9·7) |
| Chronic bronchitis | 2656 (2·3) | 25 (5·2) |
| Emphysema | 952 (0·8) | 14 (2·9) |
| Acute bronchiolitis | 15 (0·0009) | 0 (0·0) |
| Total | 116 355 | 485 |

**Legend:** ICD = International Classification of Diseases.

**Notes:** airway disease was defined using ICD-9-CM ICD-10-CA codes. A person required one hospital visit, or three of more physician visits within one year, with one or more of the above conditions to be considered to have airway disease.

**Table S3. Cohort characteristics stratified by respiratory tuberculosis status among people immigrating to British Columbia, Canada, 1985-2015**

| **Characteristic** | **Controls**  **N (%)** | **Respiratory TB**  **N (%)** | **SMD** |
| --- | --- | --- | --- |
| N | 1 004 187 | 1141 |  |
| Airway disease | 116 355 (11·6) | 485 (42·5) | 0·742 |
| Follow-up time-at-risk (mean (SD)) | 11·1 (7·3) | 12·6 (7·6) | 0·199 |
| Sex = Male | 486 680 (48·5) | 663 (58·1) | 0·194 |
| Age, years (mean (SD)) | 32·7 (16·6) | 43·6 (19·1) | 0·607 |
| Neighbourhood income quintile |  |  | 0·214 |
| Highest 20% | 146 670 (14·6) | 99 (8·7) |  |
| Middle-High 20% | 143 795 (14·3) | 145 (12·7) |  |
| Middle 20% | 182 888 (18·2) | 201 (17·6) |  |
| Low-Middle 20% | 230 440 (22·9) | 285 (25·0) |  |
| Lowest 20% | 300 394 (29·9) | 411 (36·0) |  |
| Education level |  |  | 0·312 |
| None/Unknown | 127 787 (12·7) | 152 (13·3) |  |
| Secondary or less | 431 531 (43·0) | 642 (56·3) |  |
| Trade/diploma | 184 793 (18·4) | 169 (14·8) |  |
| University degree | 260 076 (25·9) | 178 (15·6) |  |
| Immigration class |  |  | 0·540 |
| Economic | 594 081 (59·2) | 379 (33·2) |  |
| Family | 303 110 (30·2) | 570 (50·0) |  |
| Refugee | 27 799 (2·8) | 58 (5·1) |  |
| Other | 79 197 (7·9) | 134 (11·7) |  |
| TB incidence rate in country of origin at time of immigration |  |  | 0·806 |
| <100 per 100 000 pop. | 418 376 (41·7) | 121 (10·6) |  |
| 100 to <200 per 100 000 pop. | 327 249 (32·6) | 419 (36·7) |  |
| 200 to <300 per 100 000 pop. | 142 613 (14·2) | 342 (30·0) |  |
| 300+ per 100 000 pop. | 115 949 (11·5) | 259 (22·7) |  |
| Year of immigration (mean (SD)) | 14·6 (7·1) | 10·5 (6·2) | 0·613 |
| Charlson comorbidity score (mean (SD)) | 0.05 (0·3) | 0·13 (0·6) | 0·167 |
| Ethanol dependence | 1488 (0·1) | 6 (0·5) | 0·065 |
| Substance dependence | 1732 (0·2) | 5 (0·4) | 0·048 |
| Psychosis | 2319 (0·2) | 11 (1·0) | 0·095 |
| Depression | 42 286 (4·2) | 67 (5·9) | 0·076 |
| Personal health risk proxy variable | 77 416 (7·7) | 200 (17·5) | 0·299 |

**Legend:** Asterisk (*) indicates suppressed value due to cell size <5, including cross-suppression of nearest value, due to privacy legislation in British Columbia; SD = standard deviation; TB = tuberculosis.

**Table S4. Effect measure modification of airway disease risk by respiratory tuberculosis, modified by key covariates among people immigrating to British Columbia, Canada, 1985-2015**

| **Effect modifier** | **N** | **Adjusted HR**  **(TB vs controls)** | **95% CI** |
| --- | --- | --- | --- |
| Age group | - | - | - |
| <40 years | 702 821 | 2.05 | 1·76 – 2·38 |
| 40+ years | 302 507 | 2·48 | 2·22 – 2·77 |
| Sex |  |  |  |
| Male | 487 343 | 2·13 | 1·89 – 2·33 |
| Female | 517 985 | 2·03 | 1·78 – 2·33 |
| Immigration class |  |  |  |
| Economic | 594 460 | 2·49 | 2·08 – 2·98 |
| Family | 303 680 | 1·95 | 1·74 – 2·20 |
| Refugee | 27 857 | 1·88 | 1·42 – 2·47 |
| Other | 79 331 | 2·49 | 1·73 – 3·59 |
| Education level |  |  |  |
| University degree | 260 254 | 2·57 | 1·98 – 3·34 |
| Trade/diploma | 184 962 | 2·33 | 1·81 – 3·00 |
| Secondary or less | 432 173 | 2·17 | 1·93 – 2·45 |
| None/unknown | 127 939 | 1·61 | 1·31 – 1·97 |
| Neighbourhood income quintile |  |  |  |
| Highest 20% | 146 769 | 1·99 | 1·42 – 2·80 |
| Middle-High 20% | 143 940 | 2·86 | 2·24 – 3·67 |
| Middle 20% | 183 089 | 2·34 | 1·91 – 2·87 |
| Low-Middle 20% | 230 725 | 2·09 | 1·76 – 2·43 |
| Lowest 20% | 300 805 | 1·80 | 1·54 – 2·09 |
| TB incidence in country of birth |  |  |  |
| <200 per 100 000 | 746 165 | 2·57 | 2·24 – 2·94 |
| 200+ per 100 000 | 259 163 | 1·79 | 1·59 – 2·01 |
| Charlson comorbidity score |  |  |  |
| 0 | 963 174 | 2·25 | 2·05 – 2·47 |
| 1 | 35 943 | 1·04 | 0·72 – 1·50 |
| 2+ | 6211 | 1·52 | 0·82 – 2·84 |
| Depression |  |  |  |
| No | 962 975 | 1·63 | 1·60 – 1·67 |
| Yes | 42 353 | 1·12 | 0·80 – 1·57 |
| Personal health risk proxy variable |  |  |  |
| No | 927 712 | 2·15 | 1·95 – 2·37 |
| Yes | 77 616 | 1·63 | 1·33 – 2·00 |

**Legend:** BC = British Columbia; CI = confidence interval; ETOH = ethanol dependence; HR = hazard ratio; N = analytic sample size; SD = standard deviation; TB = tuberculosis.

**Notes:** Cox proportional hazards regression was used with the following covariates included to adjust for potential confounding in the relationship between respiratory TB and airway disease: age at index, sex, income quintile at index, educational qualification upon immigration, immigration class, TB incidence in country of birth, weighted Charlson comorbidity score, year of immigration, ETOH, substance dependence, psychoses, and depression.

**Table S5.** **Cohort characteristics of people diagnosed with respiratory tuberculosis, stratified by airway disease or censoring (outcome) status among people immigrating to British Columbia, Canada, 1985-2015**

|  | **Censored**  **N (%)** | **Airway disease**  **N (%)** | **p-value ^a^** |
| --- | --- | --- | --- |
| Respiratory TB patients | 656 | 485 |  |
| Follow-up time (mean (SD)) | 16·08 (6·93) | 7·96 (5·89) | <0·001 |
| Sex= Male | 389 (59·3) | 274 (56·5) | 0·374 |
| Age, years (mean (SD)) | 38·07 (17·84) | 48·67 (19·05) | <0·001 |
| Neighbourhood income quintile |  |  | 0·138 |
| Highest 20% | 66 (10·1) | 33 (6·8) |  |
| Middle-High 20% | 82 (12·5) | 63 (13·0) |  |
| Middle 20% | 108 (16·5) | 93 (19·2) |  |
| Low-Middle 20% | 154 (23·5) | 131 (27·0) |  |
| Lowest 20% | 246 (37·5) | 165 (34·0) |  |
| Education level | - | - | <0·001 |
| None/Unknown | 57 (8·7) | 95 (19·6) |  |
| Secondary or less | 368 (56·1) | 274 (56·5) |  |
| Trade/diploma | 109 (16·6) | 60 (12·4) |  |
| University degree | 122 (18·6) | 56 (11·5) |  |
| Immigration class |  |  | <0·001 |
| Economic | 259 (39·5) | 120 (24·7) |  |
| Family | 285 (43·4) | 285 (58·8) |  |
| Other | 29 (4·4) | 29 (6·0) |  |
| Refugee | 83 (12·7) | 51 (10.5) |  |
| TB incidence rate in country of origin at time of immigration |  |  | <0.001 |
| <100 per 100 000 pop· | 88 (13·4) | 33 (6·8) |  |
| 100 to <200 per 100 000 pop. | 140 (21·3) | 202 (41·6) |  |
| 200 to <300 per 100 000 pop. | 240 (36·6) | 179 (36·9) |  |
| 300+ per 100 000 pop. | 188 (28·7) | 71 (14·6) |  |
| Year of immigration (mean (SD)) | 11·81 (6·45) | 8·72 (5·40) | <0·001 |
| Charlson comorbidity score (mean (SD)) | 0·12 (0·57) | 0·13 (0·57) | 0·761 |
| Personal health risk proxy variable | 109 (16·6) | 91 (18·8) | 0·387 |

**Legend:** BC = British Columbia; CI = confidence interval; HR = hazard ratio; N = analytic sample size; SD = standard deviation; TB = tuberculosis.

**Notes:** ^a^ p-values are derived from Chi-square tests for goodness-of-fit for categorical variables, and t-test for equality of means for continuous variables.
